# Proteomic Analysis of Biomineralization Proteins in the Shell Plates and Spicules of Chiton *Acanthochitona rubrolineata*

**DOI:** 10.3390/ani16172651

**Published:** 2026-08-24

**Authors:** Jinzhe Du, Jinzhe Zhang, Jingliang Huang, Xue Liu, Chuang Liu

**Affiliations:** 1 School of Marine Science and Engineering, Qingdao Agriculture University, Qingdao 266109, China; 2 Jiangsu Province Engineering Research Center for Marine Bio-Resources Sustainable Utilization, College of Oceanography, Hohai University, Nanjing 210024, China; 3 Southern Marine Science and Engineering Guangdong Laboratory (Guangzhou), Guangzhou 511458, China

**Keywords:** spicule proteome, polyplacophora, molluscan shell, matrix proteins, shell formation

## Abstract

Chitons, ancient polyplacophoran mollusks, are ideal models for studying biomineralization evolution due to their conserved morphology since the Cambrian. This study investigates the matrix proteins in shell plates and spicules of *Acanthochitona rubrolineata* using liquid chromatography–tandem mass spectrometry. By extracting proteins from 30 individuals and using proteomic method, we identified 26 soluble proteins and 22 insoluble proteins in the shell plates and 25 insoluble proteins, and found domains such as von Willebrand factor type A, chitin-binding, ferritin, and cadherin. These domains, prevalent in molluscan biominerals, suggest conserved roles in organic matrix formation. Despite genomic dynamism, the conservation of key domains across species highlights a core biomineralization mechanism. Notably, eight of the shell proteins and eight of the spicule proteins were homologous between *A. rubrolineata* and chiton *Acanthopleura loochooana*, indicating functional conservation. Phylogenetic analysis further supported the evolutionary significance of these domains in chitons. The study advances understanding of biomineralization in Polyplacophora, emphasizing the interplay between morphological stasis and molecular evolution.

## Simple Summary

Chitons are ancient marine mollusks with relative conservation of the body plan for millions of years, making them valuable for studying how organisms build their hard parts through biomineralization. In this study, we investigated the proteins in the shell plates and spicules (small calcareous spines) of the chiton *Acanthochitona rubrolineata* using proteomics. We found 28 proteins (26 soluble proteins and 22 insoluble proteins) in the shell plates and 25 insoluble proteins in the spicules, many of which contain common functional domains such as von Willebrand factor type A, chitin-binding, ferritin, and cadherin. These domains are also found in other mollusks, suggesting that they play fundamental roles in biomineral formation. When we compared our results with those from another chiton species, we found that several proteins were shared, indicating that key biomineralization mechanisms are conserved despite genetic differences. This work helps us better understand how chitons build their protective armor and provides insights into the evolution of biomineralization across mollusks and other animal groups.

## 1. Introduction

Biomineralization denotes the biologically controlled process in which inorganic minerals are deposited under the regulation of biomacromolecules. Mineralized products, primarily found in hard tissues such as bones, teeth, and shells, serve critical functions including protection and physical support [1]. Notably, molluscan shells exhibit diverse and intricate three-dimensional architectures and are intensively studied in the biomineralization field [2]. The shell formation process is precisely controlled by macromolecules (particularly matrix proteins) embedded in the mineral phase, which are present in small amounts (less than 5% *w*/*w*) [3].

Currently, studies on shell mineralization are mainly focused on bivalves and gastropods in the mollusk phylum, while biomineralization in other classes has received little attention [4,5]. Polyplacophora, as a conserved and ancient class of mollusks, was separated from other lineages during the Late Cambrian period (about 500 million years ago), occupying a unique phylogenetic position within Mollusca [6]. *Acanthochitona rubrolineata* is a widely distributed chiton species, inhabiting coastal areas of China, the Korean Peninsula, and Japan [7]. This species was selected for the present study because it is particularly abundant in the intertidal zone of the Yellow Sea, where it is readily accessible; its relatively large body size facilitates the collection of sufficient shell and spicule material for protein extraction. Moreover, it belongs to the order Chitonida, which enables a meaningful comparative analysis with our previously published proteomic data from *Acanthopleura loochooana* (also Chitonida but from a different family). There are eight articulated calcareous shell plates on its dorsal surface, surrounded by a girdle of various calcareous structures, including scales, spines, and needles arranged in bundles or clusters called spicules. Chitons usually inhabit rocks within the intertidal zone, scraping algae from rocks using their radula [7]. Chitons can be traced back to the Late Cambrian period, and remarkably, their fossil morphology has remained virtually unchanged since that time [8]. This exceptional morphological stasis provides insights into the early evolution and origin of mollusks, making chitons an important node for understanding molluscan phylogeny. Thus, studying the proteins within the biominerals of *A. rubrolineata* and revealing their homology with those from other chitons may enable the evaluation of their degree of evolutionary conservatism.

Research on chiton biomineralization has primarily focused on teeth owing to their exceptional magnetic properties [9,10,11], whereas investigations of their shells and spicules have largely been restricted to microstructural analyses [12,13], with proteomic studies continuing to be substantially unexplored. Chiton shell represents the only known group of extant mollusks to integrate aesthetes (small sensory structures) within the outermost layer of their shells [13], and their eight shell plates and spicules serve as flexible armor against predators [13]. The investigation of the formation of chiton shell plates and spicules would help us better understand the evolution of molluscan minerals. Recently, we have reported the first shell proteome [14] and spicule proteome [15] in the chiton *Acanthopleura loochooana*, which lives in the southeast coastal areas of China. In this study, we extracted proteins from the shells and spicules of *A. rubrolineata*, a species living in the northern part of China coastal areas, and applied proteomic analysis to identify protein components, especially biomineralization-related proteins.

## 2. Materials and Methods

### 2.1. Animals

A total of 50 adult individuals of the chiton *A. rubrolineata* with a body length of 2.5–3.2 cm were collected from the waters of Taipingjiao Park, Qingdao, Shandong Province, China (36°03′57.78″ N 120°22′9.80″ E, collected in July 2023). No permit is needed to collect this chiton in China.

### 2.2. Extraction of Matrix Proteins from Chiton Spicules and Shells

The protein extraction procedure was performed according to our previous studies [14,15]. All experimental procedures, including sample handling and extraction, were carried out at 4 °C to maintain protein stability and minimize degradation. Thirty individuals of *A. rubrolineata* were dissected with a scalpel to collect the shell plates and spicules. The specimens were pooled into a single biological sample to obtain sufficient protein mass for LC-MS/MS analysis, given the known low abundance of matrix proteins in chiton biominerals (<5% *w*/*w*). The two SDS-PAGE lanes represent technical replicates (duplicate loading of the same pooled extract), not biological replicates. Shells and spicules were separately immersed in a 5% NaOH solution for 24 h to remove the soft tissues and were subsequently air-dried and ground into powder using a multifunctional pulverizer (yielding 5.5 g of powder of shell plates and 1.0 g of powder of spicules). Next, the powder was decalcified in 0.8 M EDTA (pH 8.0) for 48 h (for spicules) or 60 h (for shell plates) with constant stirring using a magnetic stirrer (700 rpm). The mass ratio of powder to EDTA was 5. During the extraction, the protease inhibitor AEBSF (1 mM) was added to the solution. For EDTA-soluble protein extraction, the solution was centrifuged, and the supernatant was collected and transferred to an ultrafiltration membrane (MWCO 3000 Da, VAKE, JiNan, China). The supernatant was dialyzed in 1000 mL of deionized water for 48 h, and the water was replaced twice during the process. The dialyzed solution was then transferred to ultrafiltration tubes (MWCO 3000 Da, Millipore, Burlington, MA, USA) and centrifuged at 5000 *g* for 1 h (15 min intervals) to remove salts and concentrate the EDTA-soluble protein. The pellets obtained from the previous step (referred to as EDTA-insoluble protein) were rinsed thoroughly with deionized water and then treated with a denaturing solution (30 mM Tris-HCl, pH 8.0, 1% SDS, 10 mM DTT) at 100 °C for 30 min, with shaking every 5 min. The extracted proteins were quantified by the Bicinchoninic Acid method. The shell-soluble, shell-insoluble, and spicule-insoluble proteins were 0.21, 0.35, and 0.65 mg·mL^−1^. Next, 10 μL of protein solution was mixed with 2 μL of loading buffer. Then, 12% SDS-PAGE gel electrophoresis was performed at 140 volts for 60 min. The gel was stained with Coomassie Brilliant Blue.

### 2.3. Liquid Chromatography–Tandem Mass Spectrometry (LC/MS-MS)

The LC/MS-MS process was based on a previously established protocol for chiton shells. The extracted proteins were stained with Coomassie Brilliant Blue, and the entire protein lane was excised into two sections (two technical replicates), which were completely destained by washing with 50 μL of 50 mM NH_4_HCO_3_/acetonitrile (50/50) mixture at 37 °C for 30 min. Subsequent reduction was performed using 50 μL of 10 mM DTT in 50 mM NH_4_HCO_3_ at 57 °C for 1 h, immediately followed by alkylation in the dark with 50 μL of 100 mM iodoacetamide for 30 min at room temperature. The gel pieces were dried in acetonitrile and then treated with 0.4 μg of trypsin (Proteomics Grade, Sigma, St. Louis, MO, USA) in 10 μL of 50 mM NH_4_HCO_3_ for 12 h at 37 °C. The resulting suspension was treated with 50 μL of 1% formic acid at 30 °C for 30 min under agitation. Finally, the digests were lyophilized and resuspended in 30 μL of 0.1% trifluoroacetic acid and 4% acetonitrile for subsequent LC-MS/MS analysis.

For LC-MS/MS, 5 μL of the samples was injected into the LTQ Orbitrap Velos mass spectrometer coupled with the Dionex U-3000 Rapid Separation nano-LC system (Thermo Scientific, Waltham, MA, USA). Peptide separation was achieved on a Thermo Scientific PepMap™ RSLC C18 column (5 µm particle size, 100 Å pore size). The flow rate was set at 300 nL/min with a linear gradient of 5–80% acetonitrile in 0.1% formic acid over 120 min. The MS data were acquired automatically by Thermo Proteome Discoverer 1.4 (Thermo Scientific, Waltham, MA, USA), following an MS survey scan over *m*/*z* 350–1500 at a resolution of 60,000 for full scan and 2000 for MS/MS measurements. The MS peaks were searched against the translated proteome database of *A. rubrolineata* (downloaded from NCBI, registry number PRJNA515172; the ORF is predicted using TBtools [16] and the total entries are 165866 unigenes; also see supporting fasta files) using a Mascot 2.1 search engine with carbamidomethylated cysteine as a fixed modification, while oxidized methionine and tryptophan were considered as variable modifications. The peptide MS and MS/MS mass tolerances were set to 0.5 Da, with a minimum requirement of two matched unique peptides for valid identification. Finally, proteins with Mascot scores over 40 and peptide spectrum matches (PSMs) of at least 2 were retained for further analysis.

### 2.4. Bioinformatic Analysis

The domain arrangement, signal peptide, and internal repeat were predicted using SmartPage (http://smart.embl-heidelberg.de/). BLAST (2.17.0) analysis between the sequences of *A. rubrolineata* and the proteome database of *A. loochooana* was performed with TBtools-II using an e-value cutoff of 1 × 10^−5^ under default settings [16]. Multiple sequence alignment was conducted by MEGA11 using the MUSCLE algorithm. Phylogenetic analysis was also performed using MEGA11 with the neighbor-joining method to construct gene trees. Protein sequences for constructing gene trees were acquired from NCBI (accessed on 1 July 2024) or MolluscaDB2.0 (http://mgbase.qnlm.ac/home) [17]. The multiple sequence alignment results were visualized using ESPript 3.x (https://espript.ibcp.fr/ESPript/cgi-bin/ESPript.cgi, accessed on 13 July 2026) and Jalview software 2.11.5.1.

### 2.5. Scanning Electron Microscopy (SEM)

The spicules were dissected and air-dried. The morphology of spicules was observed using the Quanta 250 FEG (FEI, Morristown, NJ, USA), and the surface elements were detected using the Oxford INCA-X-MAX50 energy dispersive spectrometer (High Wycombe, UK).

## 3. Results and Discussion

### 3.1. SDS-PAGE of Matrix Proteins from Chiton Shells and Spicules

**Figure 1:**
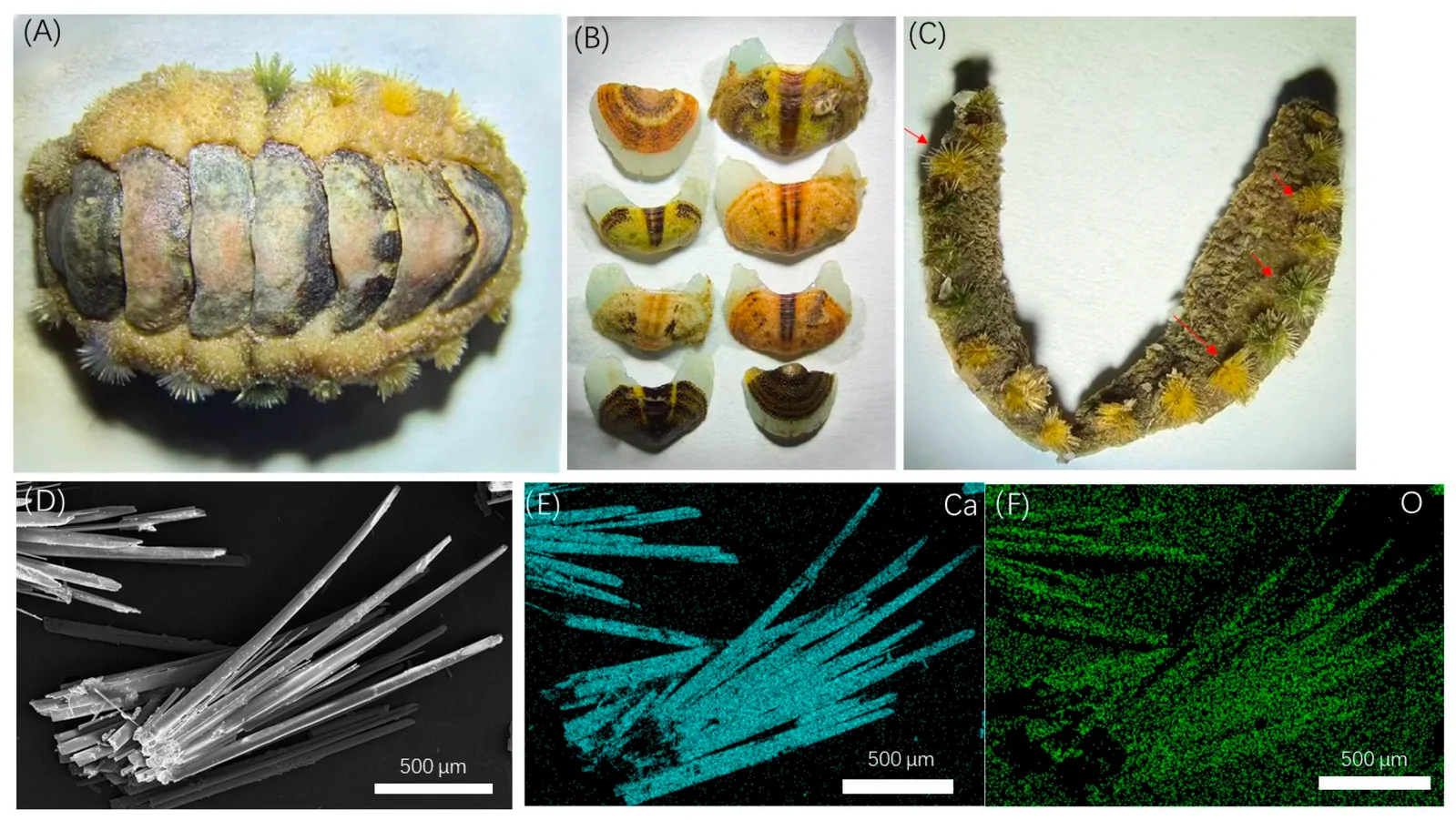
The anatomy of *A. rubrolineata*. Images of *A. rubrolineata* under a stereomicroscope (**A**); shell plates of *A. rubrolineata* (**B**); spicules of *A. rubrolineata* (red arrows) (**C**); SEM (**D**) and EDS analysis (**E**,**F**) of *A. rubrolineata* spicules; Ca and O element distribution is shown.

The chiton body exhibits an elongated oval morphology, measuring 2–3 cm in length (Figure 1A). The dorsal surface is covered by eight shell plates (Figure 1B), which are surrounded by a girdle that bears dense arrays of rod-shaped calcareous spicules (Figure 1C). Living *A. rubrolineata* has spicule projections on the girdle ring and eight shell plates on the dorsal surface, which were visible with the naked eye or under a stereomicroscope. SEM showed that the spicules were 1.02 ± 0.15 mm in length and 38.7 ± 5.2 μm in diameter (Figure 1D). Elemental analysis showed that calcium and oxygen were enriched in the spicules, indicating that they were made of calcium-based materials and possibly calcium carbonate (Figure 1E,F).

**Figure 2:**
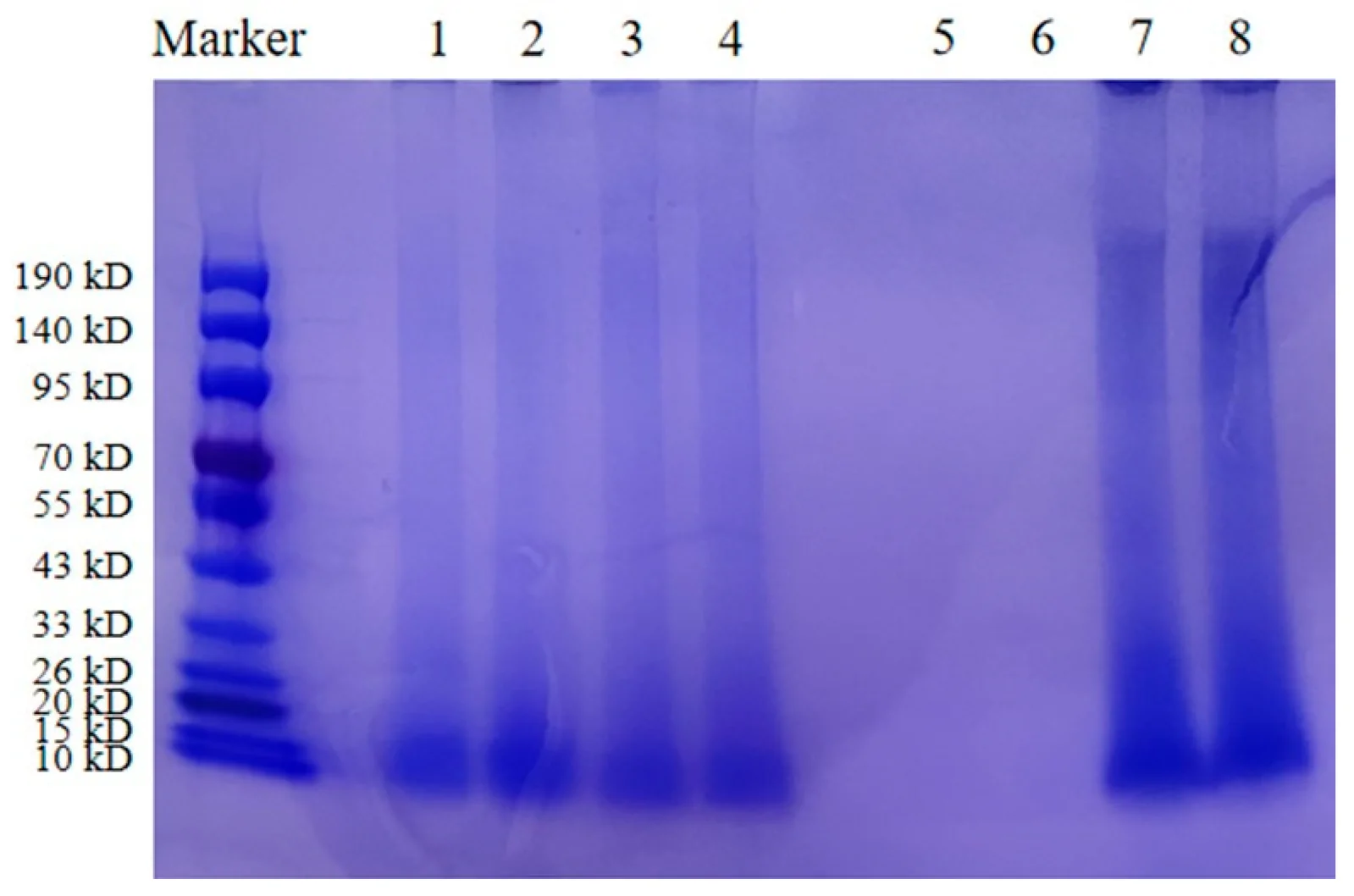
SDS-PAGE of extracted proteins from shell plates and spicules in *A. rubrolineata.* Lanes 1 and 2 represent shell matrix EDTA-soluble proteins; lanes 3 and 4 represent shell matrix EDTA-insoluble proteins; lanes 5 and 6 represent spicule EDTA-soluble proteins; lanes 7 and 8 represent spicule EDTA-insoluble proteins.

The shell plates and spicules of thirty chitons were dissected and pulverized separately for protein extraction. SDS-PAGE analysis revealed that the EDTA-soluble and EDTA-insoluble proteins from the shell matrix of *A. rubrolineata* were distributed across the entire molecular weight range (Figure 2). In contrast, the spicule EDTA-insoluble proteins exhibited a prominent band between 95 and 190 kDa, while no detectable bands were observed for the spicule EDTA-soluble proteins (Figure 2), likely due to low abundance or degradation (the soluble protein content in the spicules was likely too low to be detected by CBB staining, possibly due to their highly crystalline mineral phase or low matrix protein abundance). The phylogenetic tree of *A. rubrolineata* and *A. loochooana* suggests that the two species belong to the same order Chitonida [18]. *A. rubrolineata* and *A. loochooana* belong to the *Chitonidea* family and *Crytoplacoidea* family, respectively. Notably, unlike protein bands extracted from tissues or cells, the CBB staining band of shell plates and spicules appeared blurred, which is common for proteins extracted from other molluscan shells [19,20].

### 3.2. Protein Repertoires in the Shell and Spicule of A. rubrolineata

**Figure 3:**
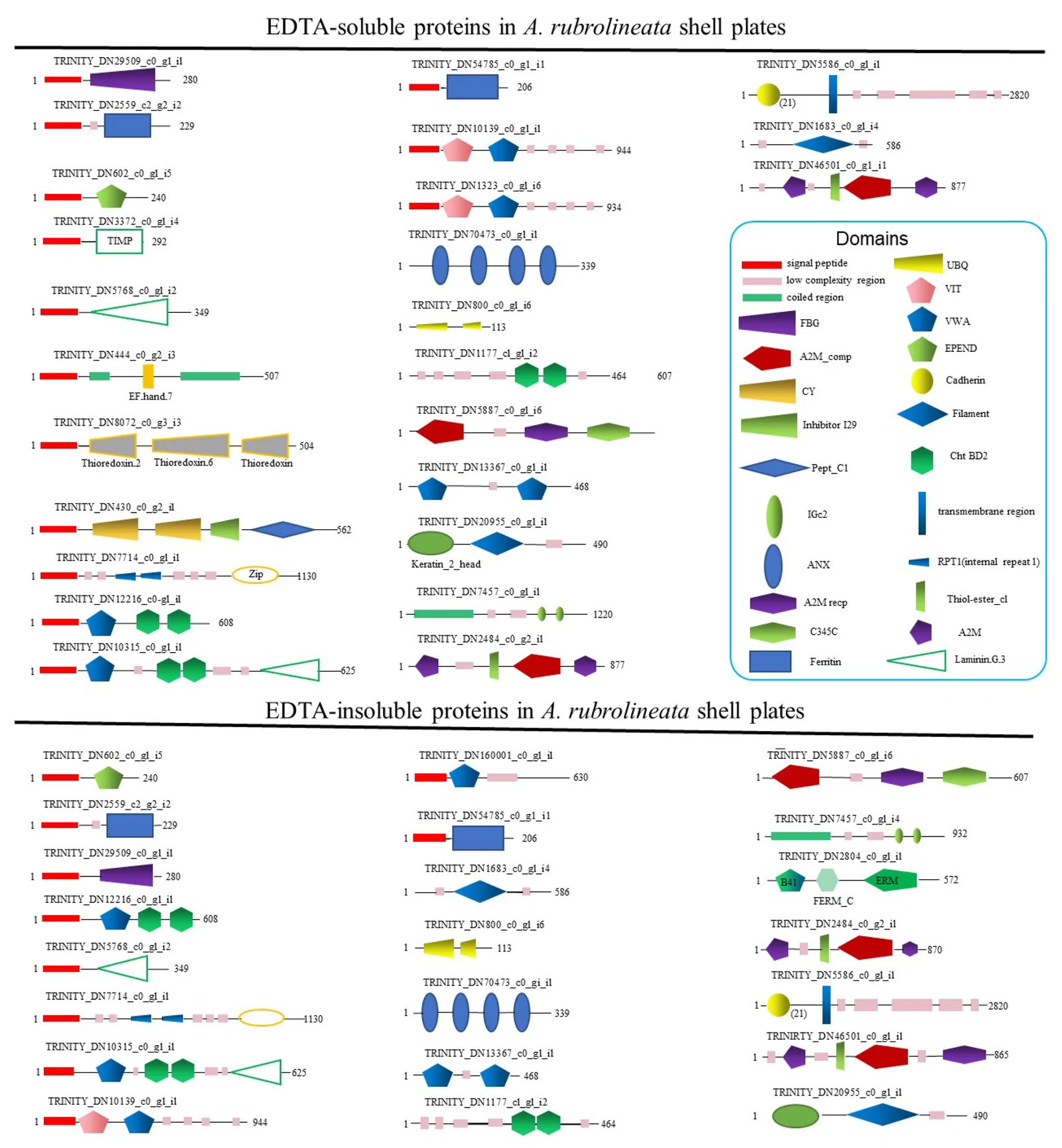
Domain arrangements of the EDTA-soluble and -insoluble proteins in *A. rubrolineata* shell plates. The legends of the domains are shown in the inset.

**Figure 4:**
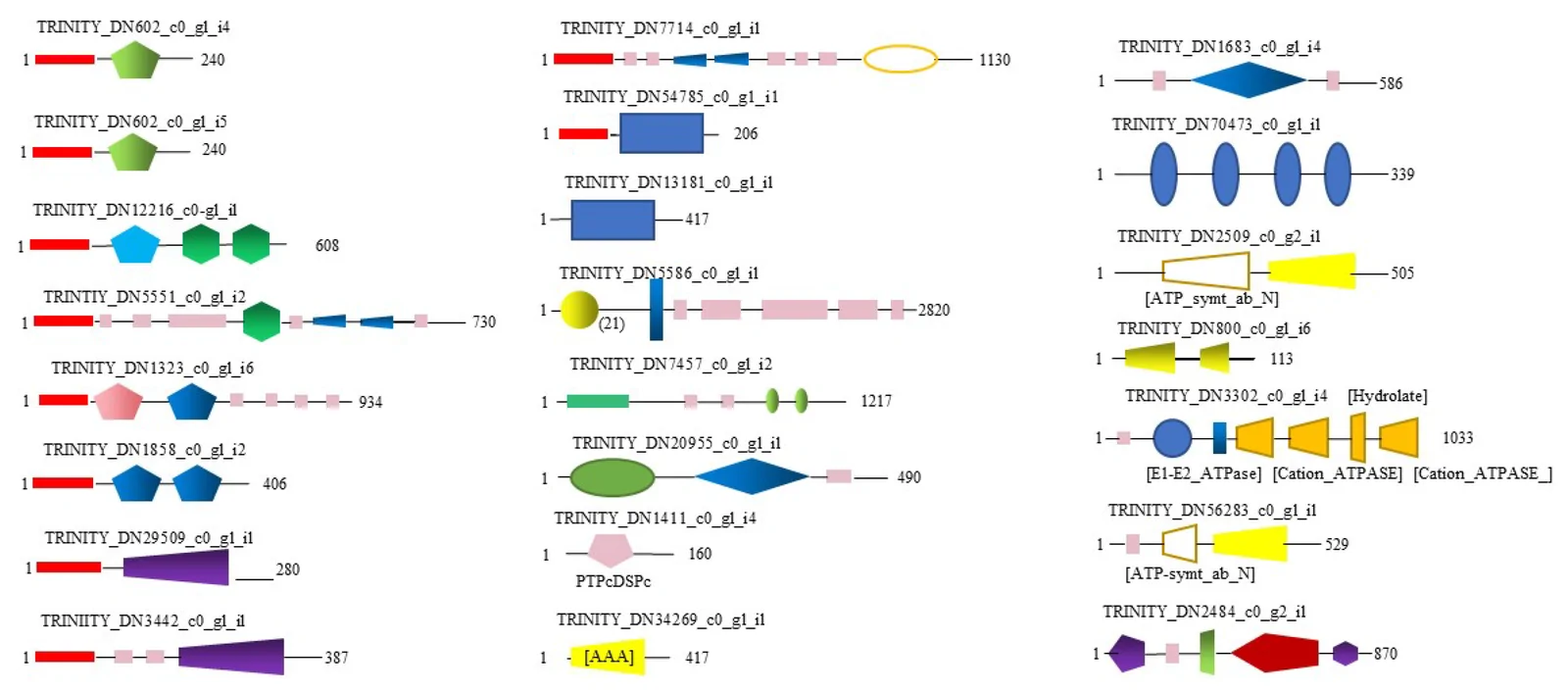
Domain arrangements of EDTA-insoluble proteins in *A. rubrolineata* spicules. Legends of the domains are consistent with Figure 3.

Protein compositions of the shells and spicules were analyzed using LC-MS/MS. A total of 26 shell EDTA-soluble proteins (with Mascot scores greater than 40) were characterized (Table S1), while 22 shell EDTA-insoluble proteins were identified (Table S2). The screening of biomineralization-related proteins was based on previous research findings, which focus on chitin-related proteins, carbonic anhydrase, immunological proteins, low-complexity regions containing proteins, and others [4,21]. Cellular and ubiquitous proteins such as histone, actin, tubulin, and myosin were not included as biomineralization proteins. Among these, eight proteins containing well-recognized biomineralization-related domains (including cadherin, vWA/ChtBD2, ferritin, filament, annexin, and ubiquitin) were shared between the soluble and insoluble shell fractions (Tables S1 and S2). Notably, however, many other shared proteins lacking these classical domains were also present in both fractions, indicating that the overall overlap between the two fractions is substantially broader than this core set. In the spicules, a total of 25 spicule EDTA-insoluble proteins were identified (Table S3). The results showed that a variety of biomineralization-related protein domains were widely distributed both in the shells and spicules of *A. rubrolineata* (Figure 3 and Figure 4), such as von Willebrand factor type A domain (vWA), chitin-binding domain (ChtBD), ferritin, and cadherin. The high percentage of protein entries with no known conserved domains implies that these proteins are likely to be unique to chitons, but the function of these proteins remains to be elucidated. The absence of a known domain may be due to an incomplete sequence, incorrect ORF prediction, or database limitations.

Domain analysis of shell and spicule proteins in *A. rubrolineata* revealed some highly conserved domains, suggesting these domains may play fundamental roles in biomineralization processes. Through domain analysis (Figure 3 and Figure 4), we characterized whether the proteins were intracellular proteins or extracellular proteins based on the presence of signal peptides. Meanwhile, multiple low-complexity regions of varying lengths were identified in some proteins.

Notably, we used 5% NaOH to clean the shell plate and spicules before EDTA treatment, which may lead to degradation or removal of some matrix proteins.

### 3.3. The Comparison of Biomineral Proteins Between A. rubrolineata and A. loochooana

**Figure 5:**
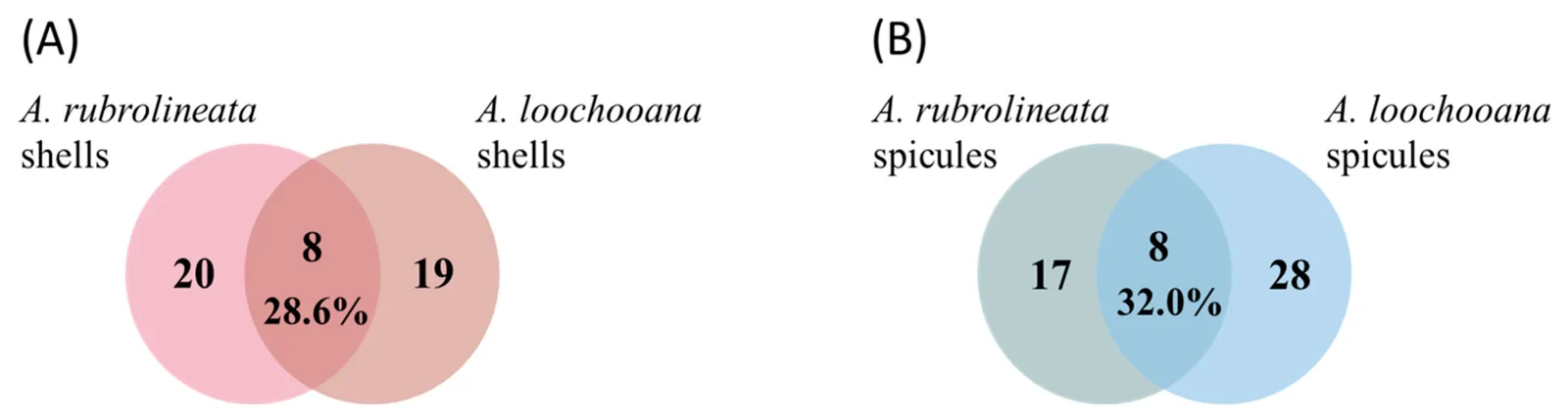
Comparison of biomineralization proteins in *A. rubrolineata* and *A. loochooana*. (**A**) Venn diagram of shared biomineralization-related proteins in the shells; (**B**) Venn diagram of shared biomineralization-related proteins in the spicules.

Given that the soluble and insoluble fractions of the shell matrix may differ in their biochemical properties and functional roles, directly combining them for cross-species comparison could potentially obscure fraction-specific differences. Nevertheless, to obtain a broad overview of the conserved molecular repertoire, we first compared the combined shell protein sets of the two chitons at the domain level. Within this framework, eight shared proteins with similar domain arrangements were identified between the shells of *A. rubrolineata* and *A. loochooana* (E-value < 1.00 × 10^−50^) (Figure 5), representing 28.6% of the total shell proteins identified in *A. rubrolineata*. Meanwhile, eight proteins with conserved domains were homologous in the spicules of two chitons (Figure 5B), comprising 32.0% of the spicule proteins in *A. rubrolineata* (Table 1 and Table 2). While the common proteins in the two chitons were less than expected, many functional domains were shared in the protein repertoires of the shells or spicules, indicating a conserved biomineralization mechanism on the domain level. Some domains were more frequently found in the biomineral-bearing proteins in both chitons, including cadherin, annexin, ferritin, vWA, ChtBD2, thioredoxin, and filament. Some of these protein domains have been extensively studied in other organisms, yet their specific functions in chitons are still unknown. Further investigation on protein sequence and structure should be conducted to elucidate their evolutionary conservation, which would provide insights into their functional roles in biomineralization.

Because the shell plates and spicules are two types of biominerals in chitons, we aimed to uncover their evolutionary connections by comparing the protein domains in the shells and spicules of *A. rubrolineata*. Domain profiling revealed that 14 protein domains were shared in the shells and spicules. Notably, the proteins containing shared domains represented 50% of the shell protein repertoire in *A. rubrolineata* (Table S4), suggesting conserved biomineralization mechanisms between these two biominerals. Previous studies have reported conserved protein domains between the shell and spicule of *A. loochooana*, indicating a shared biomineralization mechanism [15]. The present study supports the previous findings and offers additional evidence consistent with a common evolutionary origin. It should be noted that the pooling of soluble and insoluble fractions may underestimate or overestimate the true homology between species, and future comparative studies at the individual fraction level would provide a more detailed functional resolution.

Additionally, some identified proteins in the shells and spicules of *A. rubrolineata* lack known functional domains (not included in the number of overall proteins). We blasted these proteins against the *A. loochooana* protein database and identified 33 sequences retrieved with E-values. Among them, 18 proteins showing high sequence similarity were considered as homologs (E-values: 1.00 × 10^−144^ to 1.00 × 10^−6^) (Figure S1). Significantly, four proteins exhibited high evolutionary conservation in two chitons (E-value < 1.00 × 10^−100^). These results indicated that chitons have specific biomineralization proteins that are unknown so far.

### 3.4. Key Biomineralization Proteins Shared by A. rubrolineata and A. loochooana

**Figure 6:**
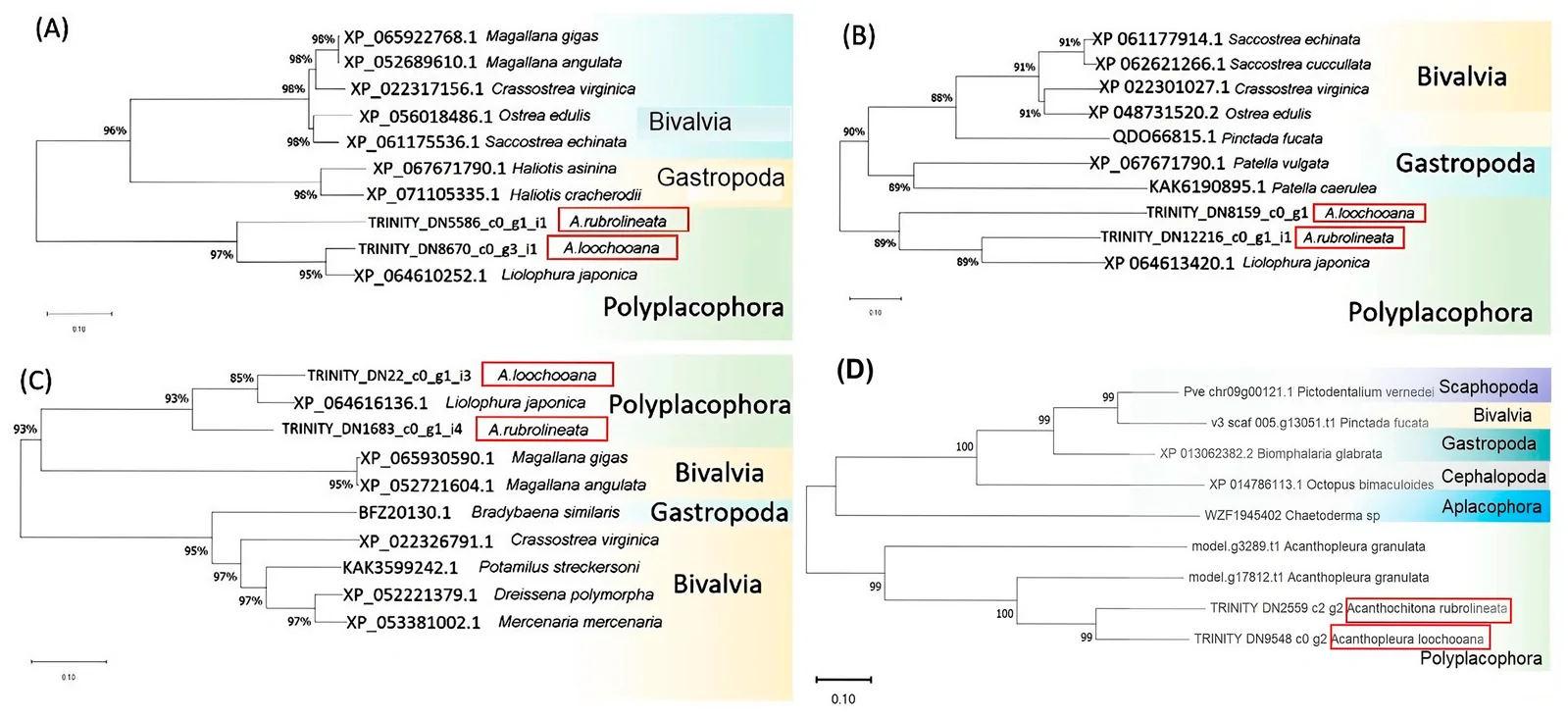
Phylogenetic trees of sequences containing domains shared by two chitons. (**A**) TRINITY_DN5586_c0_g1_i1: cadherin; (**B**) TRINITY_DN12216_c0_g1_i1: vWA/ChtBD2; (**C**) TRINITY_DN1683_c0_g1_i4: filament; (**D**) TRINITY_DN2559_c2_g2_i2: ferritin.

The currently extant chitons are classified into three well-resolved orders: Lepidopleurida, Callochitonida, and Chitonida [18]. *A. rubrolineata* and *A. loochooana* both belong to Chitonida. Interestingly, our comparison of these two species from the same order—despite their highly conserved body plans—revealed substantial divergence in their biomineralization protein repertoires. This finding suggests extensive lineage-specific protein turnover rather than a uniform protein set, highlighting the dynamic nature of molecular evolution within Chitonida. However, whether this divergence reflects an accelerated evolutionary rate would require further phylogenetic analyses with appropriate outgroups and molecular clock estimations. However, the shared proteins also point to a key molecular toolkit for biomineralization, e.g., cadherin, vWA + ChtBD2, filament, and ferritin. Therefore, we analyzed the phylogenetic tree of conserved protein sequences containing highly frequent domains (Figure 6). The results showed that these four proteins clustered together in Aculifera and formed a sister group next to other mollusks of Conchifera. Multiple alignment of four proteins indicated that they are also conserved across Mollusca (Figures S2–S5).

Cadherin is a homophilic, Ca^2+^-dependent cell adhesion glycoprotein that plays crucial roles in cell recognition, migration, tissue differentiation, embryonic development, and neural development by mechanically coupling adjacent cells and regulating various downstream signaling pathways [22]. The cadherin superfamily is a large and diverse collection of proteins with extracellular repeats of the approximately 110-residue calmodulin motif [23]. Interestingly, cadherin has been found in large abundance in the brachiopod shell matrix [24]. Previously, we have shown that cadherins are also present in shells [14] and spicules of *A. loochooana* [15], including protocadherin fat 4 and neural cadherin protein with a transmembrane domain, which may be related to neural development in the aesthetes in chiton shells [25,26]. However, the exact function of cadherins in chiton biomineralization remains unclear.

vWA and ChtBD2 domains often co-occur in biomineral proteins of both *A. rubrolineata* and *A. loochooana*. vWA is commonly found in extracellular matrix proteins that mediate adhesion through interactions [27,28]. vWA-containing proteins are prevalent in shell matrix proteins of Crassostrea gigas adult [29] and juvenile shells of blue mussels [30]. The ChtBD2 domain has a high affinity for binding chitin. Studies in the Crassostrea virginica revealed that chitin may play an essential role in shell regeneration, and two models of chitin-mediated mineralization have been proposed [31]. Previously, it has been shown that Pif protein from *Pinctada fucata*, possessing both vWA and ChtBD domains, can specifically bind aragonite crystals [32]. Since then, homologs of Pif have been identified in several different species, indicating its highly conserved nature [33,34]. In addition, the blue mussel shell protein contains both vWA and ChtBD domains and is closely related to biomineralization [34]. The co-occurrence of these two domains suggests that they may function synergistically by cross-linking the chitin fiber on the one hand while interacting with other proteins [32,35]. The finding of these two domains in chiton shells and spicules suggests that their origin may be deep in the stem lineage of all Mollusca, a hypothesis requiring broader taxonomic sampling.

Ferritin is one of the major non-heme iron storage proteins in animals, plants, and microorganisms. It consists of a mineral core of hydrated ferric oxide and a multi-subunit protein shell that encloses the former and assures its solubility in an aqueous environment. It is particularly noteworthy that among molluscan species, ferritin exhibits unique characteristics (e.g., having signal peptides) in chitons [14]. Another study on ferritin found that it may greatly contribute to shell and pearl coloration [36]. Current studies on ferritin mainly focus on the formation of chiton teeth [37,38], and its function in shell plate biomineralization remains unknown.

Filaments are prominent components of the cytoskeletons of most eukaryotic cells [39]. They play an important role in providing mechanical strength to the cell and tissue, growth and regeneration, cell survival and apoptosis, and cell migration [40]. Intermediate filaments form a complex network, concentrated in the perinuclear region, from which filaments radiate towards the cell surface [41]. The filament protein family has been identified in several mollusk species, including Mytilus coruscus, Mytilus viridis, Mytilus galloprovincialis, and C. gigas, indicating its evolutionary conservation and potential involvement in shell biomineralization processes [42]. Filament protein, as a calcium-sensitive protein, also plays a crucial role in the synthesis and transport of calcium-containing nanoparticles on the surface of the pearl layer of *Pinctada fucata* [43], which is further evidence for its crucial role in biomineralization. The presence of filament domains within chiton biomineralized tissues indicates their potential functional significance in mediating the biomineralization process.

### 3.5. Implication for Biomineralization Evolution

Recent genomics on four chitons reveals that large and dynamic genome rearrangements occurred in chitons despite their apparently similar body plans [44]. This apparent decoupling between morphological conservation and molecular/genomic plasticity raises the question of whether the biomineralization phenotype is under strong stabilizing selection, while the underlying protein-coding sequences are relatively free to diverge as long as the essential functional domains and physicochemical properties are preserved. This finding, together with our observation of extensive proteomic divergence between *A. rubrolineata* and *A. loochooana*, supports a model in which lineage-specific protein gain/loss and domain shuffling play prominent roles in chiton biomineralization evolution, even though the driving forces and precise evolutionary rates remain to be elucidated.

Beyond implications in chiton and Mollusca biomineralization evolution, the dataset generated from this study can be tentatively compared with biomineral proteomics in other phyla such as annelids [45], decapod crustaceans [46] and brachiopod [24] (Table S6). Annelid Sternaspidae is an important group of phosphate-mineralizing organisms, whose shield is composed of amorphous iron phosphate minerals [45]. Recently, it was found that in sternaspid SMPs, the vWA, EF-hand and EGF domains were the most abundant domains [45]. Compared with other biomineral proteins, sternaspid shields lacked chitin-related proteins but were rich in collagen proteins containing EGF domains, suggesting that collagen may serve as a scaffold for amorphous phosphate deposition. Decapod crustaceans, a major group of arthropods, include shrimps, crabs, lobsters, and crayfish. They have mineralized calcium carbonate as the cuticle to protect the soft tissues. Through biomineral proteomics, decapod crustaceans are found to evolve novel proteins such as chitin_bind_4 domains and cuticle_1 domains to form chitin mineralized exoskeletons [46]. However, vWA domains were absent in decapod crustaceans. Brachiopods are marine invertebrates with calcium phosphate or carbonate shells [24]. *Lingula* shells are composed of calcium phosphate. It is proposed that myosin head-containing chitin synthase and actin filaments may translate the cytoskeleton organization into an extracellular chitin scaffold. Chitinase remodels the chitin scaffold to facilitate the interaction of chitin and chitin-binding proteins. Calcium-binding proteins regulate the calcium concentration and initiate calcium phosphate deposition, together with other structural proteins, such as EGF domain-containing collagens and alanine-rich proteins [24]. This comparison indicated that biominerals from these four phyla may share certain shell matrix domains among two or three phyla, which need further comparative studies.

## 4. Conclusions

In this study, proteomic analysis was performed to reveal the key components of shell plates and spicules in the chiton *A. rubrolineata*. In total, 26 soluble proteins and 22 insoluble proteins were identified in the shell plates and 25 in the spicules. Identified proteins were enriched with vWA + ChtBD2, filament, ferritin, and cadherin domains. The function of these candidate matrix proteins should be experimentally confirmed in the future. The results may help to explore the homology and differences of biomineralization-related proteins between the two studied chiton species and provide a reference for future comparative studies involving a broader range of polyplacophoran taxa.

## Figures and Tables

**Table 1:** *A. rubrolineata* shell proteins having homologs in *A. loochooana*.

Protein Accession	Conserved Domain	E-Value	Identities
TRINITY_DN5586_c0_g1_i1	Cadherin	0	66%
TRINITY_DN1683_c0_g1_i4	Filament	0	81%
TRINITY_DN13367_c0_g1_i1	vWA	1 × 10^−97^	42%
TRINITY_DN54785_c0_g1_i1	Ferritin	2 × 10^−106^	70%
TRINITY_DN10139_c0_g1_i1	VIT/vWA	0	48%
TRINITY_DN800_c0_g1_i6	Ubiquitin	8 × 10^−53^	98%
TRINITY_DN8072_c0_g3_i3	Thioredoxin domain; Thioredoxin_2 domain; Thioredoxin_6 domain	0	84%
TRINITY_DN46501_c0_g1_i1	A2M/Thiol-ester_cl/ A2M_comp/A2M_recep	0	53%

Notes: Protein accession indicates the protein from *A. rubrolineata*; E-value and identities indicate the BLAST results from the two chitons.

**Table 2:** *A. rubrolineata* spicule proteins having homologs in *A. loochooana*.

Protein Accession	Conserved Domain	E-Value	Identities
TRINITY_DN2559_c2_g2_i2	Ferritin	6 × 10^−103^	69%
TRINITY_DN1683_c0_g1_i4	Filament	0	81%
TRINITY_DN2509_c0_g2_i1	ATP-synt_ab_N/AAA	0	94%
TRINITY_DN3442_c0_g1_i1	Fibrinogen-related domains	2 × 10^−171^	78%
TRINITY_DN5586_c0_g1_i1	Cadherin	0	66%
TRINITY_DN800_c0_g1_i6	Ubiquitin	8 × 10^−70^	98%
TRIITY_DN70473_c0_g1_i1	Annexin	1 × 10^−89^	99%
TRINITY_DN1858_c0_g1_i2	vWA	0	65%

## Data Availability

The mass spectrometry proteomics data have been deposited in the ProteomeXchange Consortium (https://proteomecentral.proteomexchange.org) via the iProX partner repository [47,48] with the dataset identifier PXD061516.

## Supplementary Materials

The following supporting information can be downloaded at https://www.mdpi.com/article/10.3390/ani16172651/s1, Figure S1: Comparison of identified proteins without a known domain between *A. rubrolineata* and *A. loochooana*; (A) Blast results of *A. rubrolineata* proteins without known domain to the *A. loochooana* database; (B–E) Alignments of the four most conserved proteins TRINITY_DN939_c0_g3_i1, TRINITY_DN7859_c0_g1_i2, TRINITY_DN926_c0_g3_i, TRINITY_DN5135_c0_g1_i4; Figure S2: Multiple alignment of cadherin across Mollusca (only partial sequences of cadherins are shown due to the large size and multiple repeats of this protein); Figure S3: Multiple alignment of vWA + ChtBD2 across Mollusca; Figure S4: Multiple alignment of filament across Mollusca; Figure S5: Multiple alignment of ferritins across Mollusca; Table S1: Soluble proteins extracted from *A. rubrolineata* shell plates; Table S2: Insoluble proteins extracted from *A. rubrolineata* shell plates; Table S3: Insoluble proteins extracted from *A. rubrolineata* spicules; Table S4: Shared biomineralization-related proteins in *A. rubrolineata* shell plates and spicules; Table S5: Number of proteins found in chitons; Table S6: Comparison of SMP domains found in four phyla.
