# Supplementary material for: Proteomic Analysis of Biomineralization Proteins in the Shell Plates and Spicules of Chiton Acanthochitona rubrolineata
Source: Animals (Basel). 2026 Aug 24;16(17):2651. doi: 10.3390/ani16172651 (PMC13564323; doi:10.3390/ani16172651)
Supplement: Supplementary file 1 [file animals-16-02651-s001.zip › animals-4413373-supplementary.pdf]

Title: Proteomic analysis of biomineralization proteins in the shell plates and spicules of chiton *Acanthochitona rubrolineata*

Jinzhe Du[a], Jinzhe Zhang[b], Jingliang Huang[c], Xue Liu[a], Chuang Liu[b]*

[a] School of Marine Science and Engineering, Qingdao Agriculture University, Qingdao 266109, China

[b] Jiangsu Province Engineering Research Center for Marine Bio-resources Sustainable Utilization, College of Oceanography, Hohai University, Nanjing 210024, China

[c] Southern Marine Science and Engineering Guangdong Laboratory (Guangzhou), Guangzhou 511458, China

E-mail: chuangliu2020@hhu.edu.cn

(A)

Protein accesion	E-Value	
TRINITY_DN7452_c0_g1_i1	1.00E-29	
TRINITY_DN464_c0_g1_i9	6.00E-75	
TRINITY_DN4296_c0_g2_i1	1.00E-07	
TRINITY_DN11134_c0_g1_i1	1.00E-47	
TRINITY_DN15451_c0_g1_i1	3.00E-21	
TRINITY_DN939_c0_g3_i1	2.00E-132	
TRINITY_DN9548_c0_g1_i2	9.00E-07	
TRINITY_DN9548_c0_g1_i3	9.00E-07	
TRINITY_DN9548_c0_g1_i4	8.00E-07	
TRINITY_DN7859_c0_g1_i2	7.00E-124	
TRINITY_DN9650_c0_g1_i1	5.00E-10	
RINITY_DN4296_c0_g2_i1	1.00E-07	
TRINITY_DN42327_c0_g1_i1	1.00E-06	
TRINITY_DN926_c0_g3_i1	4.00E-102	
TRINITY_DN1557_c0_g1_i3	3.00E-38	
TRINITY_DN3892_c0_g1_i4	1.00E-26	
TRINITY_DN2979_c0_g1_i1	4.00E-27	
TRINITY_DN5135_c0_g1_i4	1.00E-144	

E-value	Corresponding color
>1e-10	
>1e-50;<1e-10	
>1e-100;<1e-50	
<1e-100	

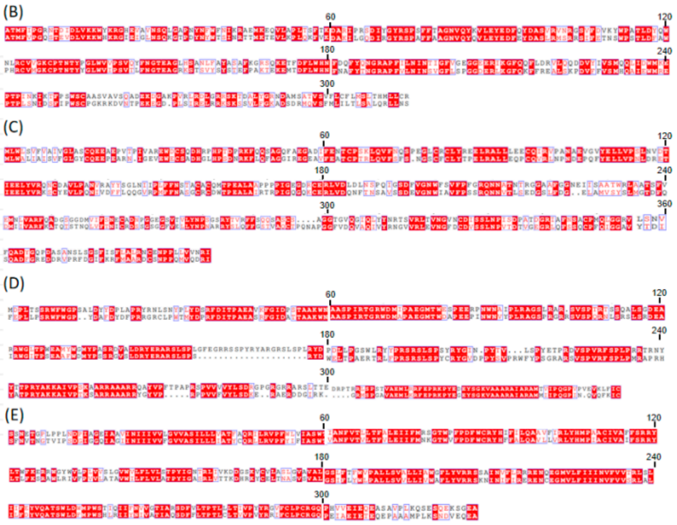

Fig.S1 Comparison of identified proteins without known domain between *A. rubrolineata* and *A. loochooana*

(A) Blast results of *A. rubrolineata* proteins without known domain to the *A. loochooana* database; (B-E) Alignments of the four most conserved proteins TRINITY_DN939_c0_g3_i1, TRINITY_DN7859_c0_g1_i2, TRINITY_DN926_c0_g3_i, TRINITY_DN5135_c0_g1_i4.

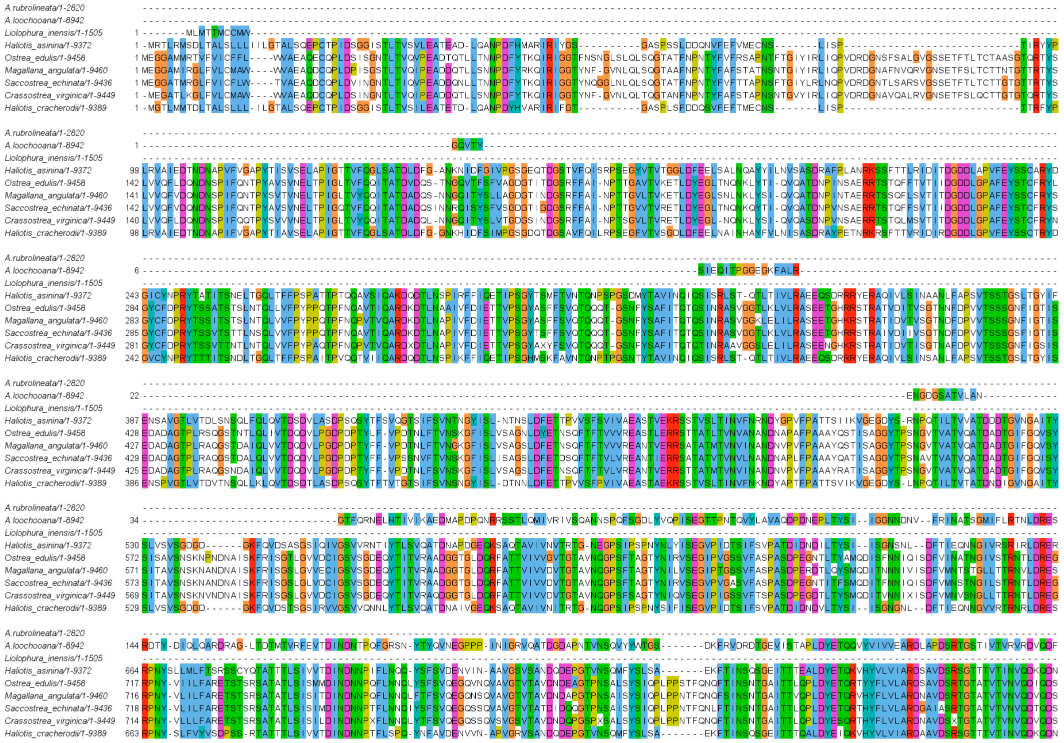

Fig. S2. Multiple alignment of Cadherin across Mollusca (only partial sequences of cadherins were shown due to the large size and multiple repeats of this protein)

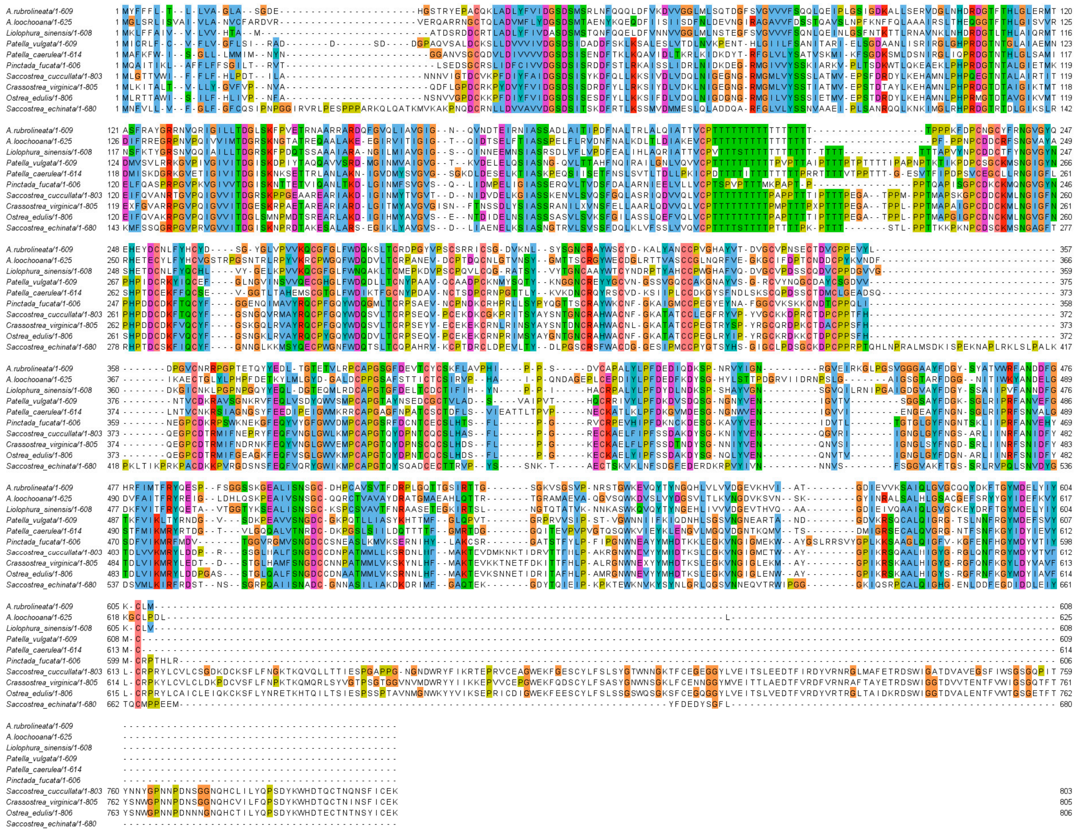

Fig. S3. Multiple alignment of vWA+ChtBD2 across Mollusca

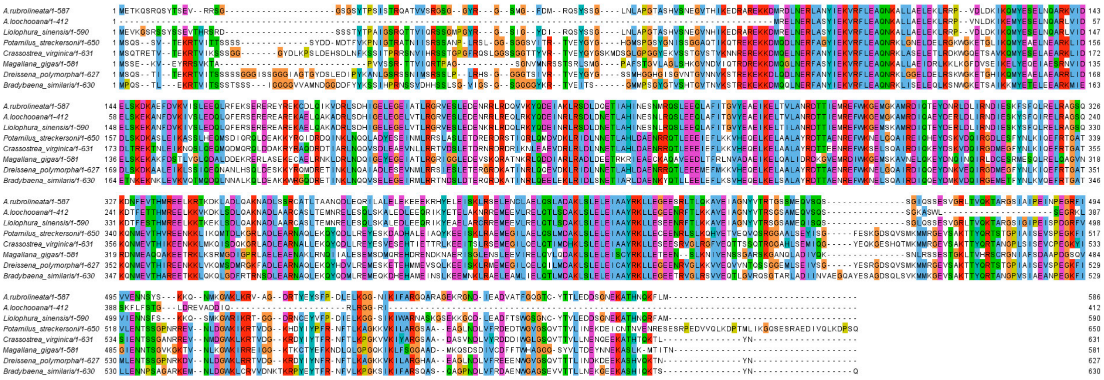

Fig. S4. Multiple alignment of Filament across Mollusca

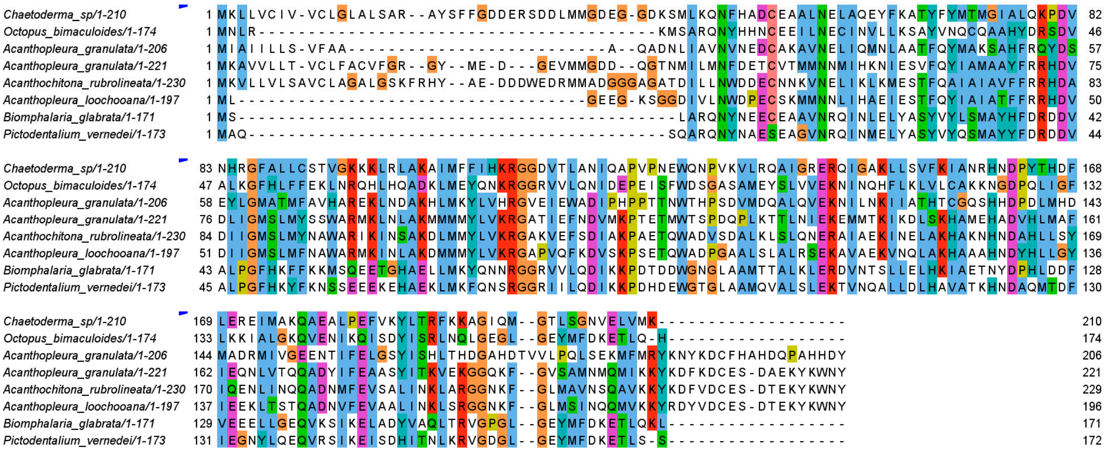

Fig. S5. Multiple alignment of ferritins across Mollusca

Table S1. Soluble proteins extracted from *A. rubrolineata* shell plates

Protein id in the transcriptome	Mascot scores	Unique peptides	PSMs	Type of domain
TRINITY_DN5586_c0_g1_i1	677.86	83	245	CA[1]
TRINITY_DN7714_c0_g1_i1	382.41	246	146	Zip[2]
TRINITY_DN12216_c0_g1_i1	245.04	15	84	vWA/ChtBD[3]
TRINITY_DN2484_c0_g2_i1	313.98	28	105	A2M/Thiolester_cl<br>A2M_comp<br>A2M_recep[4]
TRINITY_DN2559_c2_g2_i2	164.99	10	55	Ferritin
TRINITY_DN20955_c0_g1_i1	158.06	7	69	Keratin_2_head[5]
TRINITY_DN8072_c0_g3_i3	120.97	24	47	Thioredoxin<br>Thioredoxin_2<br>Thioredoxin_6
TRINITY_DN444_c0_g2_i3	74.76	8	23	EF-hand_7 [6]
TRINITY_DN3372_c0_g1_i4	43.72	8	18	TIMP[7]
TRINITY_DN29509_c0_g1_i1	93.52	8	38	FBG[8]
TRINITY_DN1177_c1_g1_i2	182.41	10	64	ChtBD2
TRINITY_DN54785_c0_g1_i1	60.75	5	17	Ferritin
TRINITY_DN602_c0_g1_i5	48.17	7	20	EPEND[9]
TRINITY_DN10315_c0_g1_i1	47.76	8	17	vWA/ChatBD2<br>Laminin_G_3[10]
TRINITY_DN46501_c0_g1_i1	69.98	13	25	A2M/Thiolester_cl<br>A2M_comp<br>A2M_recep
TRINITY_DN10139_c0_g1_i3	76.97	18	40	VIT[11]/vWA
TRINITY_DN5887_c0_g1_i6	43.33	10	19	A2M_comp<br>A2M_recep/C345[12]
TRINITY_DN7457_c0_g1_i4	51.17	20	26	IGc2[13]
TRINITY_DN13367_c0_g1_i1	42.33	11	18	vWA
TRINITY_DN5768_c0_g1_i2	48.42	5	18	Laminin_G_3
TRINITY_DN800_c0_g1_i6	54.98	4	22	UBQ[14]
TRINITY_DN1323_c0_g1_i6	50.25	14	22	vWA/VIT
TRINITY_DN70473_c0_g1_i1	81.94	16	33	ANX[15]
TRINITY_DN1683_c0_g1_i4	194.97	24	75	Filament [16]
TRINITY_DN430_c0_g2_i1	55.31	15	23	CY /Inhibitor_I29<br>Pept_C1 [17]
TRINITY_DN1117_c0_g1_i6	40.56	7	13	Pro_isomerase

Note:

1.CA: Cadherin repeats

2. Zip: zinc-regulated transporters and iron-regulated transporter-like protein

3. vWA: von Willebrand factor (vWF) type A domain; ChtBD2: Chitin-binding domain type 2

4. A2M: Alpha-2-macroglobulin family; Thiolester_cl; Alpha-macro-globulin thiol-ester bond-forming region;

5. A2M_comp; Alpha-2-macroglobulin cartilage oligomeric protein; A2M_recep: A-macroglobulin receptor
6. Keratin_2_head: Keratin type II head domain
7. TIMP: tissue inhibitor of matrix metalloproteinases
8. FBG: Fibrinogen-related domains
9. EPEND: Ependymins
10. Laminin_G_3: laminin subunit alpha 3
11. VIT: Vault protein Inter-alpha-Trypsin domain
12. C345C: Netrin C-terminal Domain
13. IGc2: Immunoglobulin C-2 Type
14. UBQ: Ubiquitin homologues
15. ANX: Annexin repeats
16. Filament: Intermediate filament protein
17.CY: Cystatin-like domain; Inhibitor_I29: Cathepsin propeptide inhibitor domain (I29); Pept_C1: Papain family cysteine protease

Table S2. Insoluble proteins extracted from *A. rubrolineata* shell plates

Protein id in the transcriptome	Mascot scores	Unique peptides	PSMs	Type of domain
TRINITY_DN5586_c0_g1_i1	1156.28	96	383	CA
TRINITY_DN7714_c0_g1_i1	613.14	40	221	Zip
TRINITY_DN12216_c0_g1_i1	521.91	20	172	vWA/ChtBD2
TRINITY_DN1683_c0_g1_i4	317.80	38	118	Filament
TRINITY_DN2484_c0_g2_i1	288.12	26	94	A2M/Thiolester_cl<br>A2M_comp/A2M_recep
TRINITY_DN2559_c2_g2_i2	183.41	10	55	Ferritin
TRINITY_DN20955_c0_g1_i1	142.46	8	57	Keratin_2_head
TRINITY_DN29509_c0_g1_i1	132.45	9	51	FBG
TRINITY_DN1177_c1_g1_i2	106.73	9	38	ChtBD2
TRINITY_DN54785_c0_g1_i1	94.60	4	27	Ferritin
TRINITY_DN602_c0_g1_i5	85.84	9	30	EPEND
TRINITY_DN10315_c0_g1_i1	81.91	12	28	vWA/ChatBD2<br>Laminin_G_3
TRINITY_DN46501_c0_g1_i1	75.43	11	24	A2M/Thiolester_cl<br>A2M_comp/A2M_recep
TRINITY_DN10139_c0_g1_i3	74.26	16	31	VIT/vWA
TRINITY_DN5887_c0_g1_i6	73.35	13	24	A2M_comp/A2M_recep<br>C345C
TRINITY_DN7457_c0_g1_i4	56.40	14	24	IGc2
TRINITY_DN16001_c0_g1_i1	53.46	9	23	vWA
TRINITY_DN5768_c0_g1_i2	52.51	5	19	Laminin_G_3
TRINITY_DN2804_c0_g1_i1	50.04	14	26	B41<br>FERM_C<br>ERM[1]
TRINITY_DN800_c0_g1_i6	47.46	4	15	UBQ
TRINITY_DN13367_c0_g1_i1	44.79	13	16	vWA
TRINITY_DN70473_c0_g1_i1	42.21	9	14	ANX

Note:

1. B41: Band 4.1 homologues; FERM_C: FERM C-terminal PH-like domain; ERM: ezrin, radixin and moesin

Table S3. Insoluble proteins extracted from *A. rubrolineata* spicules

Protein id in the transcriptome	Mascot scores	Unique peptides	PSMs	Type of domain
TRINITY_DN5551_c0_g1_i2	265.67	24	97	ChtBD2
TRINITY_DN2559_c2_g2 i2	244.01	14	63	Ferritin
TRINITY_DN602_c0_g1_i5	231.27	11	79	EPEND
TRINITY_DN7714_c0_g1_i1	153.03	21	56	Zip
TRINITY_DN20955_c0_g1_i1	123.16	7	48	Keratin_2_head<br>Filament
TRINITY_DN3442_c0_g1_i1	119.59	9	48	FBG
TRINITY_DN5586_c0_g1_i1	109.19	29	44	CA
TRINITY_DN909_c0_g1_i1	106.79	22	39	vWD
TRINITY_DN2509_c0_g2_i1	77.90	6	27	ATPsynt_ab_N<br>AAA[1]
TRINITY_DN1683_c0_g1_i4	97.16	18	37	Filament
TRINITY_DN1411_c0_g1_i4	76.34	10	20	PTPc_DSPc[2]
TRINITY_DN29509_c0_g1_i1	68.92	6	27	FBG
TRINITY_DN1858_c0_g1_i2	65.75	12	24	vWA
TRINITY_DN7457_c0_g1_i2	64.86	14	27	IGc2
TRINITY_DN54785_c0_g1_i1	56.59	3	14	Ferritin
TRINITY_DN34269_c0_g1_i1	55.07	1	19	AAA
TRINITY_DN12216_c0_g1_i1	52.97	8	16	vWA/ChtBD2
TRINITY_DN2484_c0_g2_i1	52.80	14	17	A2M/Thiolester_cl<br>A2M_comp/A2M_recep
TRINITY_DN602_c0_g1_i4	49.11	4	18	EPEND
TRINITY_DN70473_c0_g1_i1	49.05	8	17	ANX
TRINITY_DN13181_c0_g1_i1	44.74	6	14	Ferritin
TRINITY_DN1323_c0_g1_i6	42.30	12	18	VIT/vWA
TRINITY_DN800_c0_g1_i6	40.62	4	14	UBQ
TRINITY_DN56283_c0_g1_i1	42.89	1	15	ATP-synt_ab_N/AAA
TRINITY_DN3302_c0_g1_i4	40.76	7	18	Cation_ATPase_N<br>E1-E2_ATPase<br>Cation_ATPase<br>Hydrolase<br>Cation_ATPase_C

Note:

1. ATP-synt_ab_N: ATP synthase alpha and beta subunits; AAA: ATPases associated with a variety of cellular activities;

2. PTPc_DSPc: Protein tyrosine phosphatase, catalytic domain, undefined specificity

Table S4. Shared biomineralization-related proteins in *A. rubrolineata* shell plates and spicules

Protein id in the transcriptome	Type of domain
TRINITY_DN20955_c0_g1_i1	Keratin_2_head/Filament
TRINITY_DN29509_c0_g1_i1	FBG
TRINITY_DN2484_c0_g2_i1	A2M/Thiolester_cl<br>A2M_comp/A2M_recep
TRINITY_DN1323_c0_g1_i6	VIT/vWA
TRINITY_DN70473_c0_g1_i1	ANX
TRINITY_DN2559_c2_g2_i2	Ferritin
TRINITY_DN602_c0_g1_i5	EPEND
TRINITY_DN7714_c0_g1_i1	Zip
TRINITY_DN5586_c0_g1_i1	CA
TRINITY_DN800_c0_g1_i6	UBQ
TRINITY_DN1683_c0_g1_i4	Filament
TRINITY_DN13367_c0_g1_i1	vWA
TRINITY_DN7457_c0_g1_i4	IGc2
TRINITY_DN12216_c0_g1_i1	vWA/ChtBD2

Table S5. Number of proteins found in chitons

	the number of proteins
Shell soluble proteins	26
Shell insoluble proteins	22
Spicule soluble proteins	Not found
Spicule insoluble proteins	25
Shared proteins between shell soluble and insoluble fractions	20
Unique shell proteins	28

Table S6. Comparison of SMP domains found in four phyla

Domains	chitons	annelids	crustaceans	brachiopods
vWA	√	√		√
EF-hand	√	√		
EGF		√		√
chitin_bind_4			√	
cuticle_1			√	
ChtBD2	√			
chitin synthase				√
cadherin	√			√
A2M	√			
EPEND	√			
Ferritin	√			
TIMP	√			
vitellogenin			√	
tyrosinase				√
